# Transcriptome profiling and co-expression network analysis of lncRNAs and mRNAs in colorectal cancer by RNA sequencing

**DOI:** 10.1186/s12885-022-09878-6

**Published:** 2022-07-16

**Authors:** Mingjie Li, Dandan Guo, Xijun Chen, Xinxin Lu, Xiaoli Huang, Yan’an Wu

**Affiliations:** 1 Department of Clinical Laboratory, Xiang’an Hospital of Xiamen University, School of Medicine, Xiamen University, Xiamen, 361102 China; 2 Shengli Clinical Medical College of Fujian Medical University, Fujian Medical University, Fuzhou, 350001 China

**Keywords:** Colorectal cancer, lncRNA, RNA-sequencing, Co-expression

## Abstract

**Background:**

Long non-coding RNAs (lncRNAs**)** are widely involved in the pathogenesis of cancers. However, biological roles of lncRNAs in occurrence and progression of colorectal cancer (CRC) remain unclear. The current study aimed to evaluate the expression pattern of lncRNAs and messenger RNAs (mRNAs).

**Methods:**

RNA sequencing (RNA-Seq) in CRC tissues and adjacent normal tissues from 6 CRC patients was performed and functional lncRNA-mRNA co-expression network was constructed afterwards. Gene enrichment analysis was demonstrated using DAVID 6.8 tool. Reverse transcription quantitative polymerase chain reaction (RT-qPCR) was used to validate the expression pattern of differentially expressed lncRNAs. Pearson correlation analysis was applied to evaluate the relationships between selected lncRNAs and mRNAs.

**Results:**

One thousand seven hundred and sixteenth differentially expressed mRNAs and 311 differentially expressed lncRNAs were screened out. Among these, 568 mRNAs were up-regulated while 1148 mRNAs down-regulated, similarly 125 lncRNAs were up-regulated and 186 lncRNAs down-regulated. In addition, 1448 lncRNA–mRNA co-expression pairs were screened out from 940,905 candidate lncRNA-mRNA pairs. Gene enrichment analysis revealed that these lncRNA-related mRNAs are associated with cell adhesion, collagen adhesion, cell differentiation, and mainly enriched in ECM-receptor interaction and PI3K-Akt signaling pathways. Finally, RT-qPCR results verified the expression pattern of lncRNAs, as well as the relationships between lncRNAs and mRNAs in 60 pairs of CRC tissues.

**Conclusions:**

In conclusion, these results of the RNA-seq and bioinformatic analysis strongly suggested that the dysregulation of lncRNA is involved in the complicated process of CRC development, and providing important insight regarding the lncRNAs involved in CRC.

**Supplementary Information:**

The online version contains supplementary material available at 10.1186/s12885-022-09878-6.

## Background

Colorectal cancer (CRC), including colon cancer and rectal cancer, is one of the most common malignant tumors. The progression of CRC is a multi-step process and can be categorized into four stages (Dukes staging system) based on the extent of tumor invasion [1, 2]. According to the latest global cancer statistics 2018, CRC has risen to the rank third of malignant tumors and when it comes to the cancer mortality, CRC ranks second, ahead of the stomach cancer and liver cancer [3]. An upward trend in morbidity rate was observed in China, rank fourth in men and third in women [4]. In previous studies, several molecular mechanisms such as the oncogene p53, APC [5], gene methylation [6, 7] and non-coding RNA regulation [8–10] were shown to contribute to the occurrence and development of CRC. Additionally, high-throughput screening of the expression changes between CRC tumor tissues vs. adjacent normal tissues revealed a lot of diagnostic and prognostic biomarkers [11–13]. However, the comprehensive understanding of the progression and prognosis of CRC patients remains a formidable challenge due to the genetic heterogeneity and complex genomic alterations found in this cancer [14, 15].

## Methods

### Sample information

Twelve samples (harboring 6 CRC tissues and 6 paired adjacent normal tissues) used in RNA-Sequencing (RNA-Seq) were collected from six Chinese patients who were diagnosed with stage II b or IIIb CRC. The raw sequencing data is secondary analyzed, and the 6 pairs of CRC tissues were divided into two groups (group 1 and group 2, corresponding to clinical stage II and III, Table S1) based on their clinical stages. 60 pairs of CRC tissues used in expanded validation cohort were collected at Fujian Provincial Hospital from June 2015 to August 2017. We received the written informed consents from patients, and this study was reviewed and approved by the ethics committee of Fujian Provincial Hospital (No. K2012–009-01).

### Library preparation and sequencing

Total RNA was extracted from tissues with TRIzol as per the manufacturer’s protocol (Invitrogen, USA). A total of 3 μg RNA per sample was used as initial material for the RNA sample preparations. Ribosomal RNA was removed and the sequencing library was generated using Hieff NGS® MaxUp rRNA Depletion Kit (Yeasen, China) following manufacturer’s recommendations. Libraries from CRC tissue and adjacent normal tissues were analyzed on a single Genome Analyzer IIx lane (Illumina, USA) using 115 bp sequencing. Raw RNA-seq data were filtered by fastx_toolkit-0.0.14 (http://hannonlab.cshl.edu/fastx_toolkit/) according to the following criteria: 1) reads containing sequencing adaptors were removed; 2) nucleotides with a quality score lower than 20 were trimmed from the end of the sequence; 3) reads shorter than 50 were discarded; and 4) artificial reads were removed.

### Reads mapping and transcript abundance estimation

The H. sapiens reference genome (GRCh37) was downloaded in Ensemble database (Human-download DNA sequence). The original transcriptome reads sequenced were aligned against the reference genome using TopHat v1.3.1, and bam (binary SAM) file alignment results were output. The pre-built GRCh37 index was downloaded from the TopHat homepage and used as the reference genome. The aligned read files were processed by Cufflinks v1.0.3, which uses the normalized RNA-seq fragment counts to measure the relative abundances of transcripts. The unit of measurement is Fragments Per kilo-base of exon per million fragments mapped (FPKM). Confidence intervals (CI) for FPKM estimated were calculated using a Bayesian inference method.

### Differentially expressed gene testing

The downloaded Ensemble GTF file (GRCh37) was submitted to Cufflinks v2.2.1 along with the original alignment (SAM) files produced by TopHat. Cufflinks re-estimates the abundance of the transcripts listed in the GTF file using alignments from the SAM file and concurrently tests for differential expression with the default parameters. Only the comparisons with q_value less than 0.05, |log_2_FC| ≥ 1, Max FPKM (N, T) ≥1 and test status marked as “OK” in the Cufflinks output were regarded as differential expression. Meanwhile, since we hope to study the overall gene expression in colorectal cancer tissues, genes expressed separately in stage II or III respectively were excluded, which may better reflect the commonality of this sequencing.

### Functional enrichment analysis and lncRNA-mRNA co-expression network

DAVID v 6.8 is a web-based functional annotation tool. The unique lists of differentially expressed genes and all the expressed genes (FPKM> 0) were submitted as the gene list and background list, respectively. The cut-off value of the False Discovery Rate (FDR) was 0.05, and only the results from the Gene ontology analysis (GO) and Kyoto Encyclopedia of Genes and Genomes pathway analysis (KEGG) were selected as functional annotation categories. Pearson correlation analysis was used to estimate co-expression relationships between lncRNAs and mRNA. A set of co-expressed lncRNA-related genes were filtered with a Pearson coefficient threshold of 0.95 and *p* < 0.01. Cytoscape 3.2.1 tool was applied to construct the lncRNA-mRNA network.

### Validations of differentially expressed lncRNAs

The differentially expressed lncRNAs were verified by Reverse transcription quantitative polymerase chain reaction (RT-qPCR) using SYBR® Premix Ex Taq™ reagent (TAKARA, Japan) on ABI ViiA™ 7 (Applied Biosystems, USA) per the manufacturer’s instructions. The selection criteria for validation included, 1) The gene expression level was relatively high for detection; 2) The gene expression pattern was consistent in the 6 tumor tissues (all higher than/all lower than the matched normal tissues); and 3) Higher differential expression ratio in cancer/normal tissues. Primer sequences were listed in Table S2. In addition, the correlationship between MIR4435-1HG (an up-reguated lncRNA) and COL4A1, SATB2-AS1 (a down-regulated lncRNA) and SGK2, were confirmed using Pearson correlation analysis in 60 samples collected. Gene expression levels were normalized to glyceraldehyde-3-phosphate dehydrogenase (*GAPDH*). All the RT-qPCR reactions were performed in triplicate. Expression data was expressed as mean ± SD and *P* < 0.05 was considered statistically significant.

## Results

### Characterization of sequencing and mapping

**Fig. 1:**
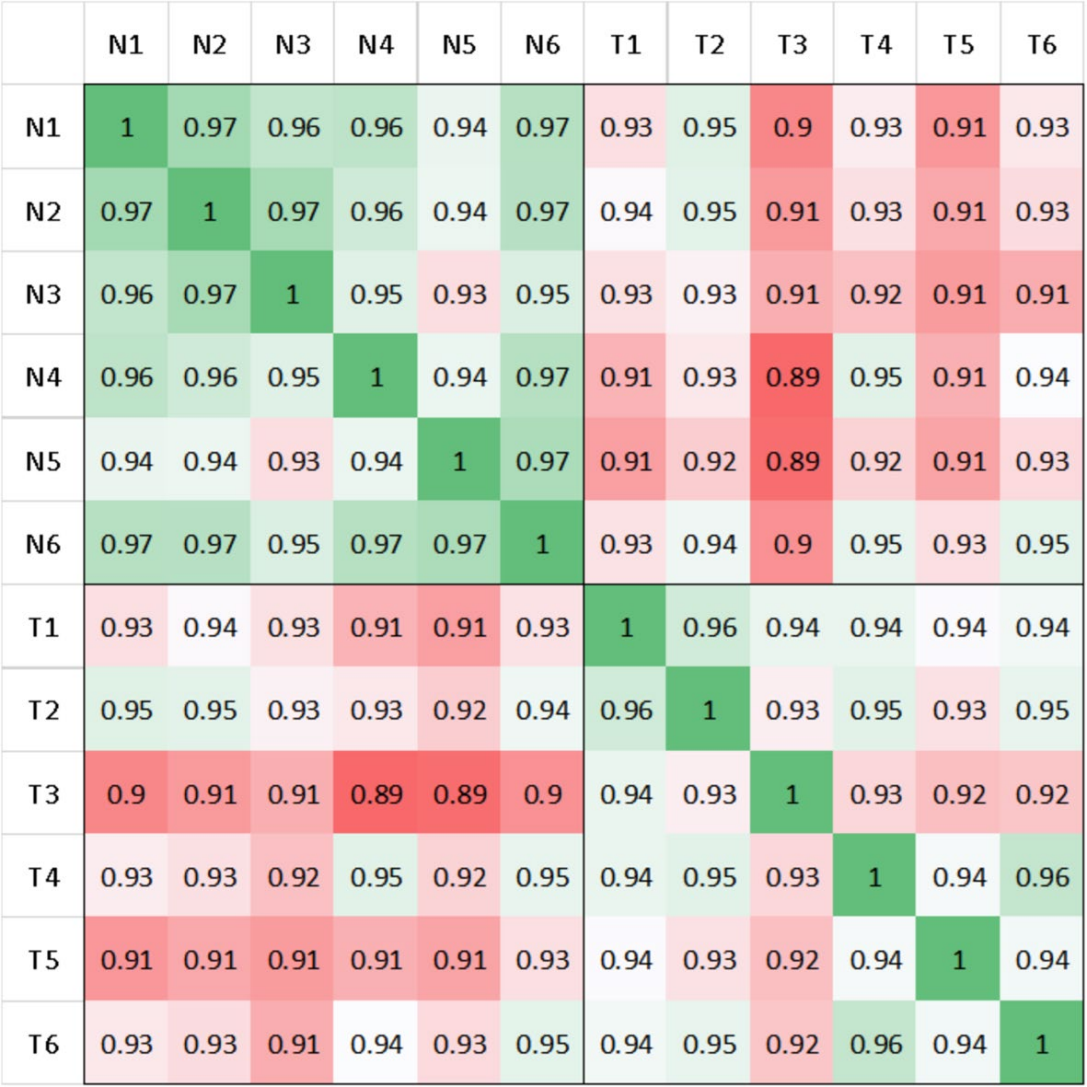
The expression correlation coefficient of 6 pair of samples. Pearson correlation analysis test was used to evaluate the correlationship between tumor and non-tumor samples. T = tumor tissues. N = normal tissues

**Fig. 2:**
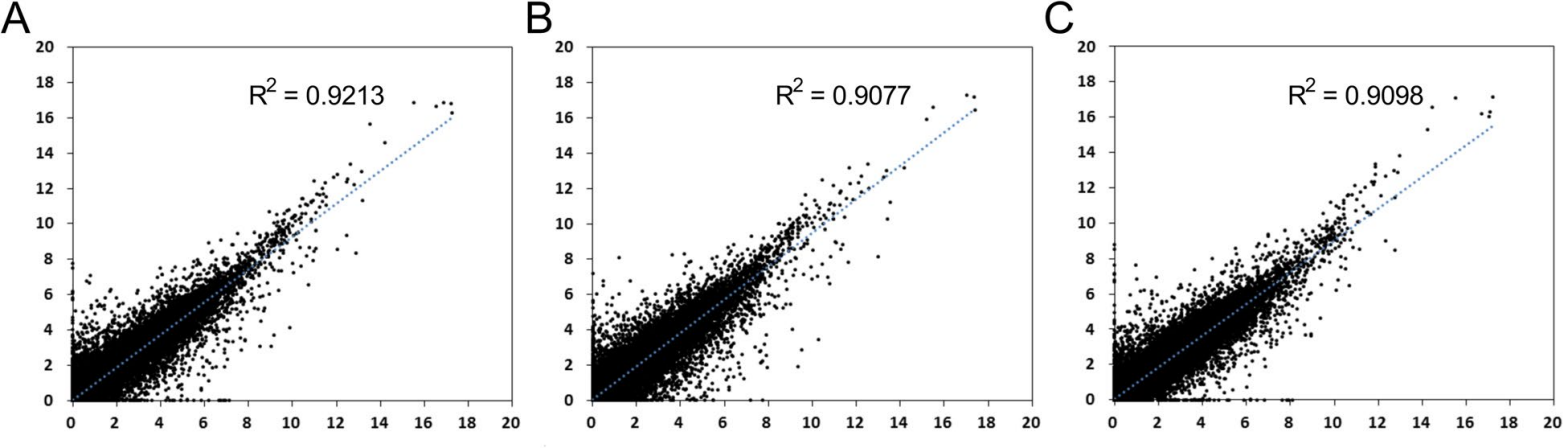
Scatter relation between CRC tissues vs. adjacent normal tissues after sample grouping. **a** Group All. **b** Group 1 (**c**) Group 2. Group All harbored 6 pair of tissues. Group 1 harbored 3 pair of tissues with clinical stage II while Group 2 with clinical stage III

All 12 samples were subjected to massively parallel paired-end cDNA sequencing. On average, 16 Gb (14.2–19.6Gb) datum were obtained from CRC tissues and adjacent normal tissues. We used TopHat tool to align the reads to the Ensemble reference human genome GRCh37. The proportion of reads that mapped to the Ensemble reference genes ranged from 82.7 to 90.9% for the twelve samples. Correlation coefficients of expression levels between different samples are shown in Fig. 1. After grouping the samples, the scatter relationship between tumor tissues and normal tissues was shown in Fig. 2. The average coverage of our sequencing depth was approximately 108(94–137) times of human transcriptome and the details of the mapping results were listed in Table 1. This sequencing received 18,489 mRNAs and 9753 lncRNAs, accounting for 89 and 70% of annotated genes (mRNA:20730, lncRNA:13869). The mRNA and lncRNA expression level of FPKM≥1 were 12,773 and 1669, accounting for 62 and 12% respectively (Table 2).

**Table 1:** The original transcriptome reads were aligned against the reference genome (GRCh37)

Sample	Total-Reads	Mapped-Reads	Total-Bases	Mapped-Bases	Mapping ratio	Coverage
s01	102,764,778	93,316,419	15,414,716,700	13,997,462,850	90.80%	108
s02	117,846,434	105,058,885	17,676,965,100	15,758,832,750	89.10%	121
s03	101,402,146	90,323,042	15,210,321,900	13,548,456,300	89.10%	104
s04	130,824,180	118,959,773	19,623,627,000	17,843,965,950	90.90%	137
s05	115,178,910	101,784,502	17,276,836,500	15,267,675,300	88.40%	117
s06	116,017,850	100,353,994	17,402,677,500	15,053,099,100	86.50%	116
s07	104,224,522	88,919,240	15,633,678,300	13,337,886,000	85.30%	103
s08	103,060,004	87,796,089	15,459,000,600	13,169,413,350	85.20%	101
s09	104,594,052	86,499,111	15,689,107,800	12,974,866,650	82.70%	100
s10	101,629,746	85,232,484	15,244,461,900	12,784,872,600	83.90%	98
s11	103,411,666	86,009,483	15,511,749,900	12,901,422,450	83.20%	99
s12	95,225,756	81,512,856	14,283,863,400	12,226,928,400	85.60%	94
Average	108,015,004	93,813,823	16,202,250,550	14,072,073,475	86.73%	108

**Table 2:** The mRNA and lncRNA expression level with FPKM> 0 and FPKM≥1

Sample	Type	lncRNA				mRNA			
		#FPKM> 0	#FPKM ≥1	%FPKM > 0	%FPKM ≥1	#FPKM > 0	#FPKM ≥1	%FPKM > 0	%FPKM ≥1
s01	N1	9399	1740	67.77%	12.55%	18,333	12,624	88.44%	60.90%
s03	N2	9759	1536	70.37%	11.08%	18,464	12,904	89.07%	62.25%
s05	N3	9867	1750	71.14%	12.62%	18,474	12,529	89.12%	60.44%
s07	N4	10,204	1744	73.57%	12.57%	18,600	12,632	89.73%	60.94%
s09	N5	9453	1613	68.16%	11.63%	18,518	13,156	89.33%	63.46%
s11	N6	9620	1587	69.36%	11.44%	18,550	13,003	89.48%	62.73%
s02	T1	9882	1640	71.25%	11.82%	18,566	12,746	89.56%	61.49%
s04	T2	9113	1657	65.71%	11.95%	18,252	12,747	88.05%	61.49%
s06	T3	9681	1899	69.80%	13.69%	18,347	12,288	88.50%	59.28%
s08	T4	10,169	1709	73.32%	12.32%	18,657	12,930	90.00%	62.37%
s10	T5	10,183	1543	73.42%	11.13%	18,633	12,848	89.88%	61.98%
s12	T6	9702	1605	69.95%	11.57%	18,471	12,870	89.10%	62.08%
Average		9753	1669	70.32%	12.03%	18,489	12,773	89.19%	61.62%

Notes: *N* normal tissues, *T* tumor tissues, *FPKM* Fragments Per kilo-base of exon per million fragments mapped

### Differentially expressed lncRNAs and mRNAs in CRC tissues

**Fig. 3:**
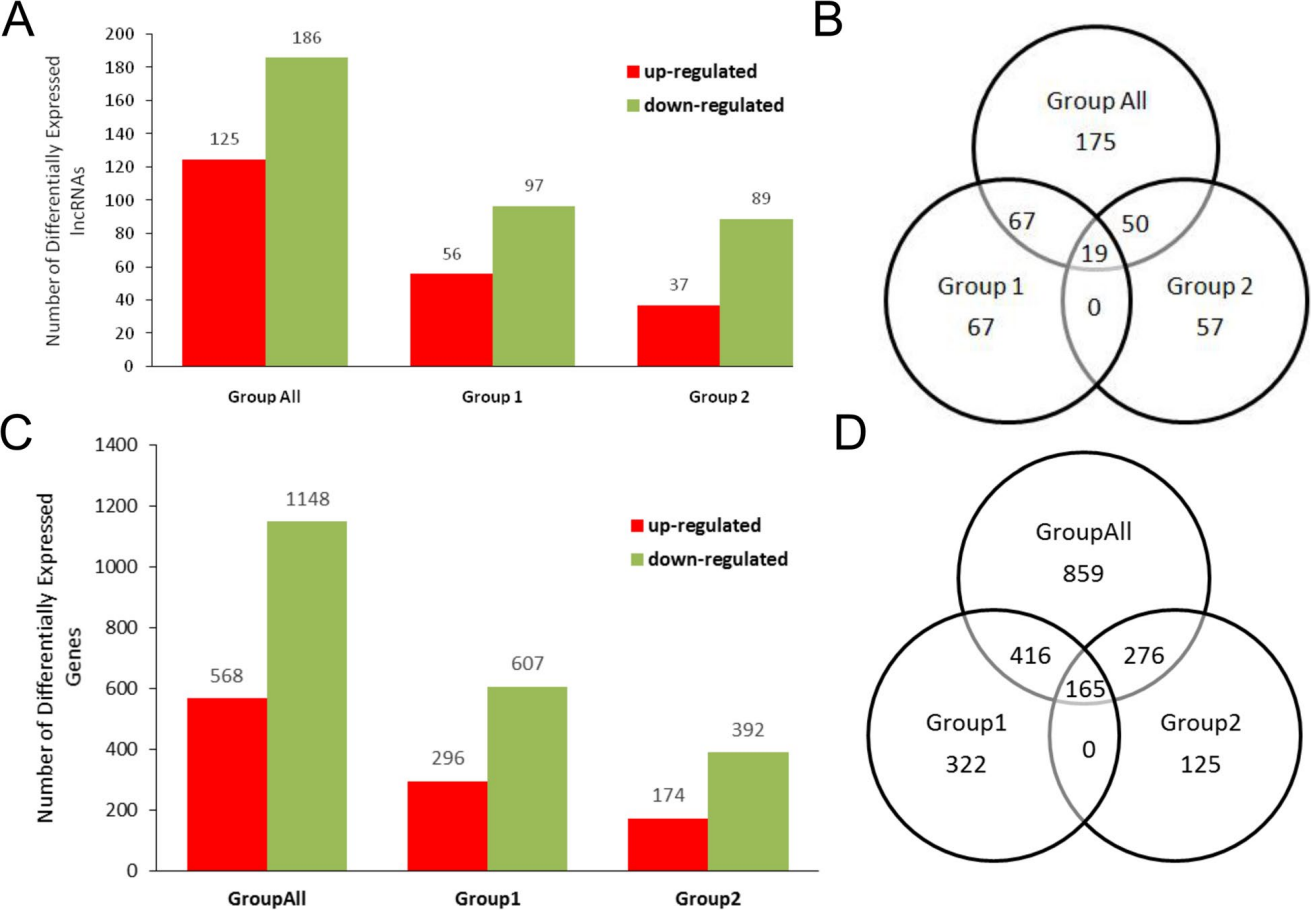
Numbers of differentially expressed genes in pre-designed groups. **a** and (**c**) Differentially expressed lncRNAs and mRNAs in three groups. **b** and (**d**) Venn diagrams of different groups of lncRNAs and mRNAs

FPKMs were calculated for normalization of the expression level of lncRNAs and mRNAs. 1716 differentially expressed mRNAs and 311 differentially expressed lncRNAs were found in 6 pairs of CRC tissues vs. adjacent normal tissues. Among these, 568 mRNAs were up-regulated while 1148 mRNAs down-regulated, similarly 125 lncRNAs were up-regulated while 186 lncRNAs down-regulated. In group I, 903 differentially expressed mRNAs and 153 differentially expressed lncRNAs were screened out. Among them, 296 mRNA were up-regulated and 607 mRNAs down-regulated while 56 lncRNAs were up-regulated and 97 lncRNAs down-regulated. In group II, 566 differentially expressed mRNAs and 126 differentially expressed lncRNAs were found. Among them, 174 mRNAs were up-regulated and 392 mRNAs down-regulated while 37 lncRNAs were up-regulated and 89 lncRNAs down-regulated (Fig. 3).

### Functional enrichment analysis and mRNA-lncRNA co-expression network

**Fig. 4:**
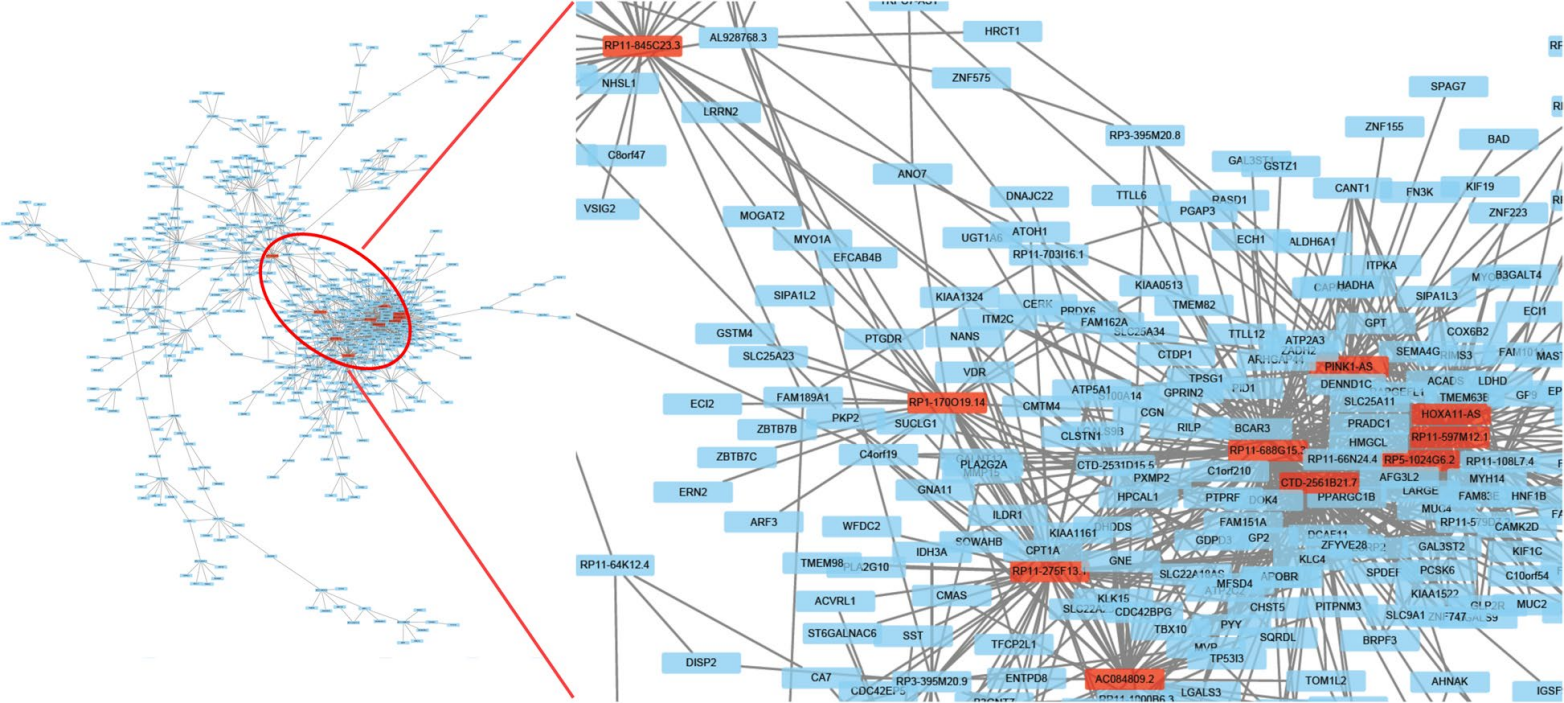
LncRNA-mRNA co-expression network. The red nodes in the network represented lncRNAs while the blue nodes were co-expressed mRNAs. LncRNAs and mRNAs with correlation coefficients greater than or equal to 0.95 were selected, and then a network was constructed using Cytoscape 3.3.1 tool

**Fig. 5:**
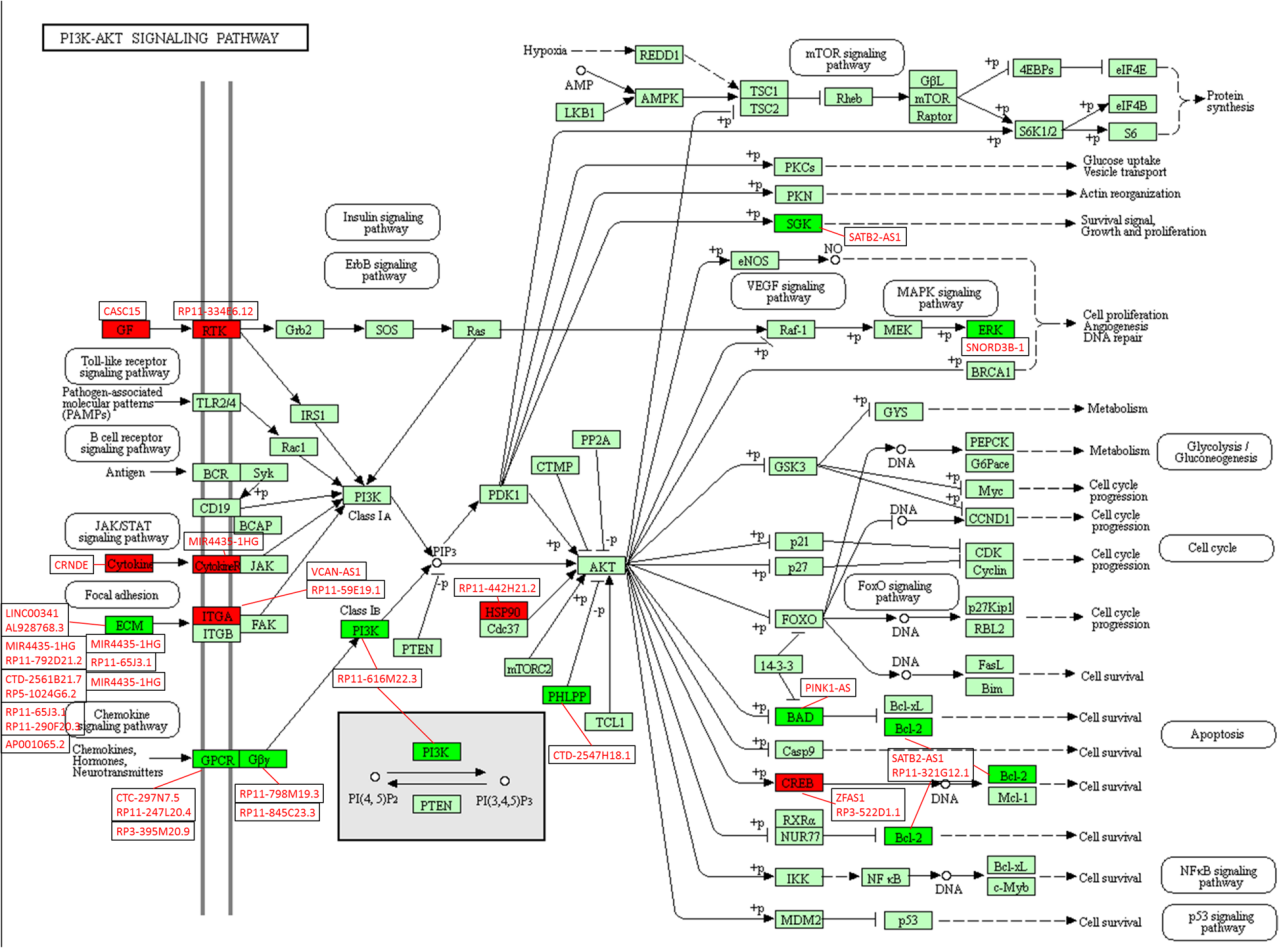
Mapping analysis for PI3K-Akt signaling pathway. Black font: mRNA. Red font: lncRNA. Red box: the up-regulated mRNAs in tumor tissues. Green box: the down-regulated mRNAs in tumor tissues. Light green box: mRNAs expressed in humans. Red line: co-expression. Note: This PI3K-Akt signaling map is derived from the KEGG online tool. [Minoru K, Miho F, Yoko S, Mari I, Mao T: KEGG: integrating viruses and cellular organisms. Nucleic Acids Res 2021, 49(D1):D545-D551]

We constructed a co-expression network of the dysregulated lncRNAs and mRNAs. 1448 lncRNA–mRNA co-expression pairs were screened out from 940,905 candidate lncRNAs and mRNAs (Fig. 4). GO analysis and KEGG revealed that these co-expression mRNAs were closely correlated with cell adhesion, collagen adhesion, cell differentiation and formation of extracellular matrix organization, and mainly enriched in fatty acid degradation, butanoate metabolism and PI3K-Akt signaling pathway (Table S3 and S4). It is public knowledge that PI3K-Akt signaling pathway had a profound effect on CRC progress. Naturally, as depicted at Fig. 5, we performed the mapping analysis for PI3K-Akt signaling pathway. According to co-expression analysis, many lncRNAs were enriched on important nodes of the PI3K/Akt signaling pathway (Fig. 5, FDR < 0.05).

### The results of RT-qPCR

**Fig. 6:**
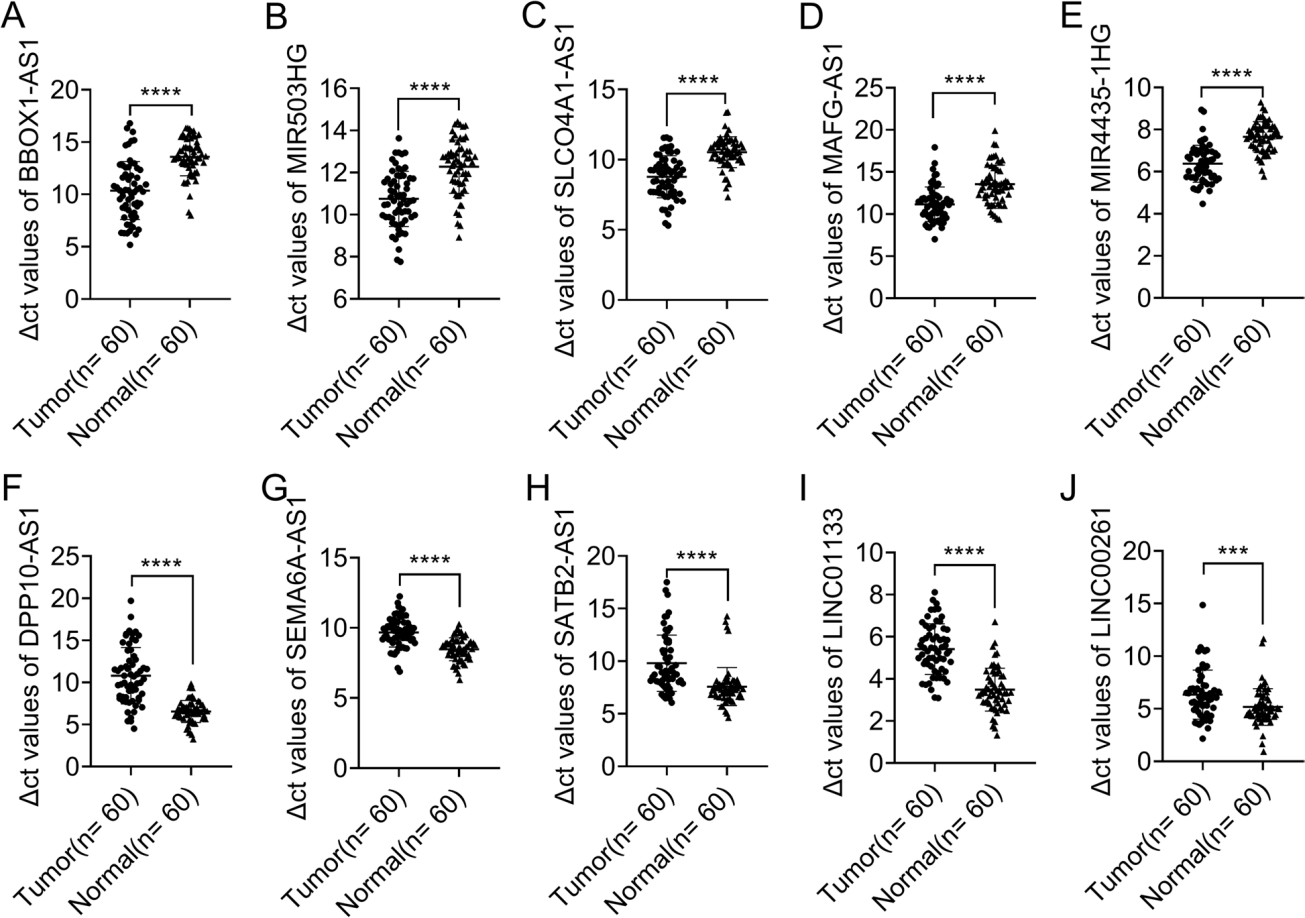
The expression values of 10 lncRNAs in an expanded 60 samples cohort. **a**, **b**, **c**, **d** and **e** The expression levels of 5 up-regulated lncRNAs. **f, g, h, i** and **j** The expression levels of 5 down-regulated lncRNAs. All the RT-qPCR tests were performed in triplicate and data were shown as means ± SD. Lower Δct values indicated higher expression. ***P < 0.001; ****P < 0.0001. *P* < 0.05 was considered statistically significant

**Fig. 7:**
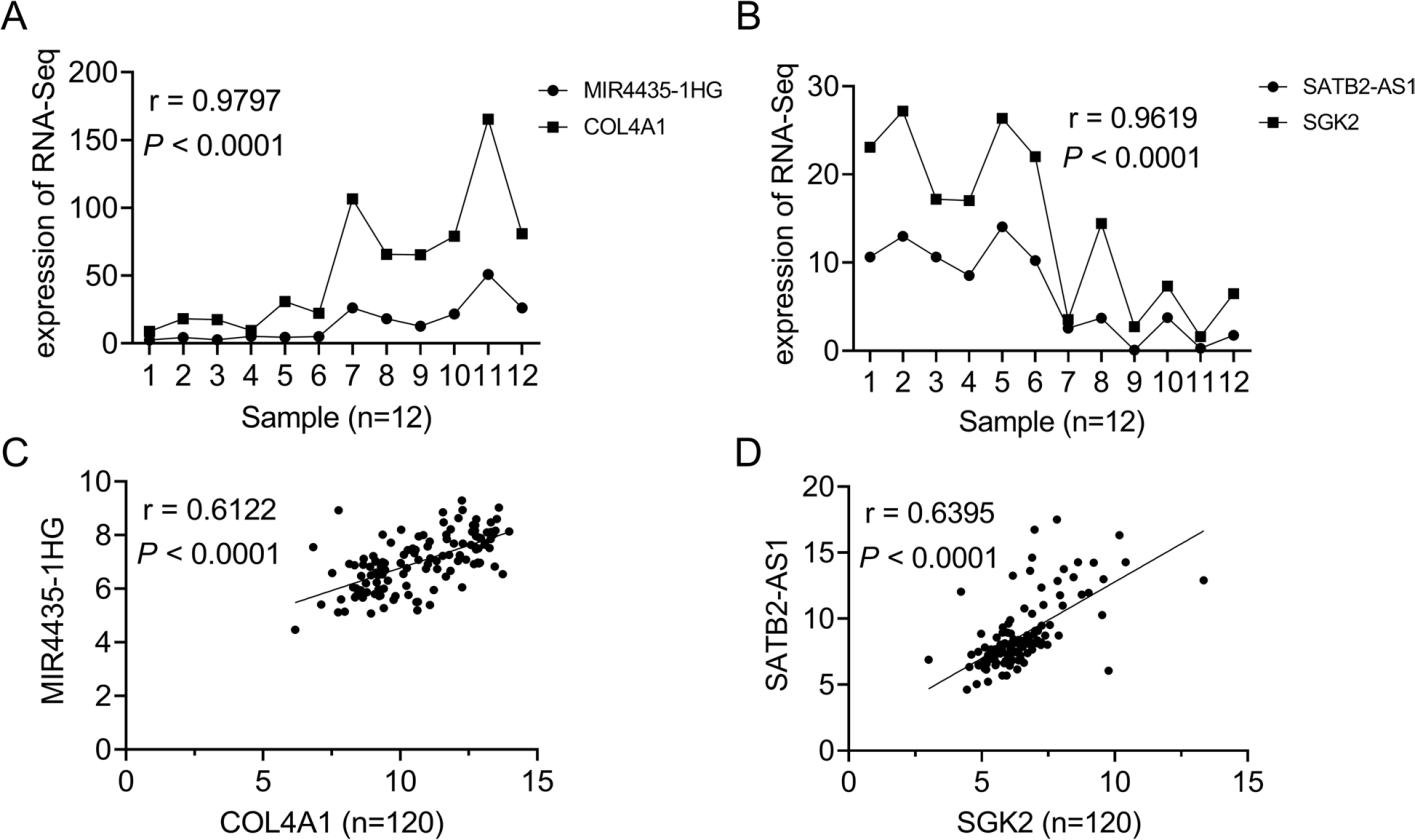
The expression correlation between selected lncRNAs and relative mRNAs. **a** and (**b**) The correlationships between *MIR4435-1HG* and *COL4A1*, *SATB2-AS1* and *SGK2* based on RNA-sequencing. **c** and (**d**) The correlationships between *MIR4435-1HG* and *COL4A1*, *SATB2-AS1* and *SGK2*, based on RT-qPCR in 120 samples. Y axes in Fig. 7A and B were the FPKM while C and D were Δct

Ten differentially expressed lncRNAs selected were as follows: RP11-1 L12.3 (BBOX1-AS1), *MIR503HG, RP11-93B14.5 (SLCO4A1-AS1), MAFG-AS1, MIR4435-1HG*, *AC066593.1 (DPP10-AS1) SATB2-AS1, CTB-118 N6.3 (SEMA6A-AS1), RP11-48O20.4 (LINC01133), LINC00261*. RT-qPCR showed that *BBOX1-AS1, MIR503HG, SLCO4A1-AS1, MAFG-AS1, MIR4435-1HG* were significantly up-regulated compared with paired normal tissues, while *DPP10-AS1, SATB2-AS1, SEMA6A-AS1, LINC01133* and *LINC00261* were significantly down-regulated compared with paired normal tissues (all *P* < 0.05, Fig. 6). Besides, the Pearson correlation analysis showed that MIR4435-1HG and SATB-AS1 were positively associated with COL4A1 and SGK2, respectively (*P* < 0.0001, *r* > 0.7; Fig. 7).

## Discussion

As one of the most malignant tumors, CRC is becoming a great social burden in the world. It was reported that there would be 18.1 million new cancer cases and 9.6 million new cancer deaths worldwide in 2018, among which CRC ranked the 4th in incidence and the 2nd in mortality, seriously endangering people’s healthy and property safety [3]. Improvement of this severe situation mainly depends on identification of biomarkers for early diagnosis and development of therapies for CRC treatment. Here, the differentially expressed mRNAs and lncRNAs were screened out by using RNA-seq for 6 pair of CRC tissues. Based on the sequencing results, differential lncRNA-mRNA co-expression network and gene list enrichment analysis revealed the potential regulatory roles of lncRNAs in the development of CRC. Finally, the expression patterns of 10 lncRNAs, as well as correlativity between selected lncRNAs and mRNAs, were detected in an expanded tissues sample set to verify the reliability of RNA-seq.

Protein-coding genes make up only 1.5–2% of the human genome, while the non-coding genes consist of almost 98%. LncRNA, a class of RNA with length more than 200 bp, is now attracting wide attention. It was once considered sort of transcriptional noises due to deletion of protein-coding regions. But now, accumulating evidences showed that lncRNAs were generally involved in many human cancers, such as glioma, gastric cancer, breast cancer, liver cancer, endometrial cancer and so on [16]. However, the underlying functional roles and mechanisms of most lncRNAs remain elusive. In last decade, a lot of lncRNAs were identified for early diagnosis and prognosis monitoring of CRC. Through the bioinformatics database and large-scale verification, Xu et al., identified the differentially expressed lncRNA-*SNHG11* as an appropriate candidate for early diagnosis of CRC patients [17]. A prognostic risk formula including three lncRNAs (*LINC01602*, *AP003555.2* and *AP006284.1*) was successfully established to evaluate the prognosis of CRC patients, these three-lncRNAs signature presented a great potential of being the independent biomarker for the prognosis of CRC patients [18]. *LINC01133* was detected down-regulated in CRC tissues and Kaplan-Meier survival analysis revealed patients with high-*LINC01133* had a better survival outcome [19]. Encouragingly, several lncRNAs mentioned above were included in our differentially expressed genes set, which also confirmed the effectiveness of the current sequencing. Based on those studies, we also hope to further analyze the impact of these dysregulated lncRNAs on early diagnosis and prognosis of CRC patients in the future.

BBOX1-AS1, an aberrant expressed anti-sense lncRNA depicted in this study, presented increasing status in CRC cell lines. Knockdown of BBOX1-AS1 inhibited the progression of CRC cell, including cell proliferation, migration, invasion and conversely promoted apoptosis of tumor cells by sponging miR-361-3p/SH2B1 regulatory axis [20]. Consistent with our study, lncRNA DPP10-AS1 was shown to be significantly decreased in CRC tumor tissues, along with changes in colon cancer stem cell properties. In vitro and in vivo studies uncovered that DPP10-AS1, worked as a tumor suppressor, inhabited proliferation, migration and invasion but facilitated apoptosis of CRC cells through the potential miR-127-3p/ADCY1 axis [21]. Another lncRNA MIR503HG in the validation set of RT-qPCR was widely known for its tumor suppressor-like role in CRC. Rescue test uncovered that overexpression of miR-107 reversed the anti-tumor effect of MIR503HG on CRC cells by potential mechanism of epithelial-mesenchymal transformation [22]. It was worth mentioning that MIR503HG was decreased in tumor tissues and cells in their study, which was contrary to the current study (Fig. 5B). On one hand, the sample set of this study might be insufficient. As was well-known, with the increase of sample size, the average expression level of the gene in the population tended to its true level. On the other hand, as mentioned above, there existed large differences in tumor heterogeneity of CRC patients, and even different parts of the same piece of tissue are expressed differently due to cell composition and genetic heterogeneity.

Drug resistance was one of the main obstacles in the therapy of CRC, and understanding of chemoresistance will greatly improve the treatment and prognosis of patients. Accumulating evidences suggested that lncRNAs might play significant roles in the chemoresistance. In vivo and vitro studies validated that lncRNA-*HAND2-AS1* inhabited the proliferation and 5-FU resistance in 5-FU-resistant CRC tumor cells [23]. Targeted lncRNA therapy has a profound prospect and may be an alternative option for CRC patients accompanied by chemotherapy resistance.

Recently, RNA-seq can be used to distinguish differences in gene expression between different time points and different groups, especially transcriptome differences between normal and tumor tissues. RNA-seq is characterized by high throughput and high repeatability and has been applied to the transcriptome study of animals and plants [24, 25]. Meanwhile, RNA-seq also provides a new means for accurate and early diagnosis of clinical diseases, and provides a feasible prospect for improving clinical treatment [26]. In the current study, RNA-seq was performed in 6 pairs of CRC tissues and differentially expressed lncRNAs were identified, which laid a foundation for our subsequent research on diagnostic biomarkers and expanded the understanding of the pathogenesis of CRC. However, the current RNA-seq technology also faced some technical difficulties. Ribosomal RNA and short-reads RNA were removed prior to sequencing in this study, and thus limiting the number of reads and the accuracy of these RNA expression levels, which might introduce potential errors and ultimately affected experimental results. Expression data could not accurately reflect gene expression levels because many genes possessed various isforms and some are rarely detected. Therefore, there still existed uncertainty about genes whose expression was significantly altered when using the cuffdiff tool. Beyond all doubt, the emergence of new technologies is inevitably in possession of disadvantages as well as advantages, and RNA-Seq does the same. But certainly, it is worth expecting that there will be more strategies to reduce these shortcomings in the future.

Based on the co-expressed mRNAs, differentially expressed lncRNAs were enriched in PI3K-Akt signaling pathway, which was known as a complicated pathway in the progress of CRC (Fig. 5). The high-frequency activation of PI3K signaling pathway has been validated sharing a close relationship with occurrence and development of CRC, contributing important values for the diagnosis and therapy of CRC [27]. Studies have confirmed that abnormal activation of PI3K occurred in the early, advanced and metastatic stages of CRC [28–30], and experiments in vivo and in vitro clearly prompted that the regulation of signal transduction system by targeted-PI3K signaling pathway had a certain improvement effect on CRC therapies [31]. In the current study, according to the construction of lncRNA-mRNA co-expression network, these lncRNAs were found to be related to cell proliferation, cycle, DNA repair event and even pathological epithelial-mesenchymal transformation, which played a crucial role in malignant metastasis and drug resistance of tumor cells. Analysis of the co-expression of lncRNA and mRNA was helpful to predict the role of lncRNA in the development of various diseases, especially in cancer, and laid a foundation for revealing their mechanism of carcinogenesis and progression. Besides, CASC15 and RP11-334E6.12 were co-expressed with GF and RTK, pivotal upstream molecule of PI3K, might make an influence on the PI3K/Akt signal and ultimately changed the biological behaviors by regulating the metabolism and proliferation of CRC cells (Fig. 5). Beyond that, as conspicuously demonstrated at Fig. 5, more than 10 aberrantly expressed lncRNAs were enriched in extracellular matrix (ECM) pathway, accompanied by PI3K/Akt pathway, might have significant impacts on tumor migration and metastasis. We firmly believed that aforementioned results would shed a light on subsequent works.

CRC is a common malignancy worldwide, with a particularly high incidence in China. The etiology, pathophysiology, and underlying molecular mechanisms of CRC remains largely unknown, and further study on roles of candidate lncRNAs needs to be fully carried out. LncRNA is becoming a research hotspot in CRC, and we hope that this study will not only expand the library of lncRNA markers in CRC, but further revealing the mystery of pathogenic mechanisms. Dysregulated lncRNAs are expected to be advisable biomarkers for early/advanced diagnosis and prognosis. We are confident that lncRNA will become the new favorite of targeted biotherapy.

## Conclusions

In summary, we comprehensively investigated the RNA profiles of CRC tumor and adjacent normal tissues through the RNA-seq technology and identified differentially expressed mRNA and lncRNAs. The abnormal expression of lncRNAs were verified and furthermore, related lncRNAs were fortunately enriched in the PI3K-Akt signaling pathway, which undoubtedly laid a solid foundation for the future work on the roles of lncRNAs in CRC.

## Supplementary Information

**Additional file 1.** RT-qPCR result s of lncRNAs and mRNAs.

**Additional file 2: Table S1. (DOCX 13 kb)**

**Additional file 3: Table S2. (DOCX 13 kb)**

**Additional file 4: Table S3. (DOCX 15 kb)**

**Additional file 5: Table S4. (DOCX 14 kb)**

## Data Availability

The datasets generated and/or analysed during the current study are publicly available in GSA database and the relevant accession number is HRA001913 (https://bigd.big.ac.cn/gsa-human/browse/HRA001913).

## Abbreviations

lncRNA: Long non-coding RNA
mRNA: messenger RNA
RNA-Seq: RNA sequencing
RT-qPCR: Reverse transcription quantitative polymerase chain reaction
CRC: Colorectal cancer
FPKM: Fragments Per kilo-base of exon per million fragments mapped
CI: Confidence intervals
FDR: False Discovery Rate
GO: Gene ontology analysis
KEGG: Kyoto Encyclopedia of Genes and Genomes pathway analysis
GAPDH: Glyceraldehyde-3-phosphate dehydrogenase

## References

[1] Cavallaro P, Bordeianou L, Stafford C, Clark J, Berger D, Cusack J, Kunitake H, Francone T, Ricciardi R (2019). Impact of single-organ metastasis to the liver or lung and genetic mutation status on prognosis in stage IV colorectal Cancer. Clin Colorectal Cancer.

[2] Hubers J, Sonnenberg A, Gopal D, Weiss J, Holobyn T, Soni A (2020). Trends in wait time for colorectal Cancer screening and diagnosis 2013-2016. Clin Transl Gastroenterol.

[3] Bray F, Ferlay J, Soerjomataram I, Siegel RL, Torre LA, Jemal A (2018). Global cancer statistics 2018: GLOBOCAN estimates of incidence and mortality worldwide for 36 cancers in 185 countries. CA Cancer J Clin.

[4] Siegel RL, Miller KD, Fedewa SA, Ahnen DJ, Meester RGS, Barzi A, Jemal A (2017). Colorectal cancer statistics, 2017. CA Cancer J Clin.

[5] Su HX, Zhou HH, Wang MY, Cheng J, Zhang SC, Hui F, Chen XZ, Liu SH, Liu QJ, Zhu ZJ (2014). Mutations of C-reactive protein (CRP) -286 SNP, APC and p53 in colorectal cancer: implication for a CRP-Wnt crosstalk. PLoS One.

[6] Ashktorab H, Brim H (2014). DNA methylation and colorectal Cancer. Curr. Colorectal Cancer Rep.

[7] Agrawal A, Murphy RF, Agrawal DK (2007). DNA methylation in breast and colorectal cancers. Modern pathology: an official journal of the United States and Canadian academy of pathology. Inc.

[8] Vishnubalaji R, Hamam R, Abdulla MH, Mohammed MA, Kassem M, Al-Obeed O, Aldahmash A, Alajez NM (2015). Genome-wide mRNA and miRNA expression profiling reveal multiple regulatory networks in colorectal cancer. Cell Death Dis.

[9] Ye J, Wu X, Wu D, Wu P, Ni C, Zhang Z, Chen Z, Qiu F, Xu J, Huang J (2013). miRNA-27b targets vascular endothelial growth factor C to inhibit tumor progression and angiogenesis in colorectal cancer. PLoS One.

[10] El-Murr N, Abidi Z, Wanherdrick K, Svrcek M, Gaub MP, Flejou JF, Hamelin R, Duval A, Lesuffleur T (2012). MiRNA genes constitute new targets for microsatellite instability in colorectal cancer. PLoS One.

[11] Lee JR, Kwon CH, Choi Y, Park HJ, Kim HS, Jo HJ, Oh N (2016). Park do Y: transcriptome analysis of paired primary colorectal carcinoma and liver metastases reveals fusion transcripts and similar gene expression profiles in primary carcinoma and liver metastases. BMC Cancer.

[12] Matouk IJ, Abbasi I, Hochberg A, Galun E, Dweik H, Akkawi M (2009). Highly upregulated in liver cancer noncoding RNA is overexpressed in hepatic colorectal metastasis. Eur J Gastroenterol Hepatol.

[13] Yang Y, Shen Z, Yan Y, Wang B, Zhang J, Shen C, Li T, Ye C, Gao Z, Peng G (2017). Long non-coding RNA GAS5 inhibits cell proliferation, induces G0/G1 arrest and apoptosis, and functions as a prognostic marker in colorectal cancer. Oncol Lett.

[14] Stratton MR, Campbell PJ, Futreal PA (2009). The cancer genome. Nature.

[15] Alves Martins BA, de Bulhoes GF, Cavalcanti IN, Martins MM, de Oliveira PG, Martins AMA (2019). Biomarkers in colorectal Cancer: the role of translational proteomics research. Front Oncol.

[16] Begolli R, Sideris N, Giakountis A (2019). LncRNAs as chromatin regulators in Cancer: from molecular function to clinical potential. Cancers.

[17] Xu W, Zhou G, Wang H, Liu Y, Chen B, Chen W, Lin C, Wu S, Gong A, Xu M (2019). Circulating lncRNA SNHG11 as a novel biomarker for early diagnosis and prognosis of colorectal cancer. Int J Cancer.

[18] Liu Y, Liu B, Jin G, Zhang J, Wang X, Feng Y, Bian Z, Fei B, Yin Y, Huang Z (2019). An integrated three-long non-coding RNA signature predicts prognosis in colorectal Cancer patients. Front Oncol.

[19] Zhang JH, Li AY, Wei N (2017). Downregulation of long non-coding RNA LINC01133 is predictive of poor prognosis in colorectal cancer patients. Eur Rev Med Pharmacol Sci.

[20] Liu J, Zhu J, Xiao Z, Wang X, Luo J: BBOX1-AS1 contributes to colorectal cancer progression by sponging hsa-miR-361-3p and targeting SH2B1. FEBS open bio 2020, Online ahead of print. 10.1002/2211-5463.12802 PMC9063435 31984680

[21] Tian H, Pan J, Fang S, Zhou C, Tian H, He J, Shen W, Meng X, Jin X, Gong Z (2021). LncRNA DPP10-AS1 promotes malignant processes through epigenetically activating its cognate gene DPP10 and predicts poor prognosis in lung cancer patients. Cancer Biol Med.

[22] Han H, Li H, Zhou J (2020). Long non-coding RNA MIR503HG inhibits the proliferation, migration and invasion of colon cancer cells via miR-107/Par4 axis. Exp Cell Res.

[23] Jiang Z, Li L, Hou Z, Liu W, Wang H, Zhou T, Li Y, Chen S (2020). LncRNA HAND2-AS1 inhibits 5-fluorouracil resistance by modulating miR-20a/PDCD4 axis in colorectal cancer. Cell Signal.

[24] Qiu W, Chen B, Greer JB, Magnuson JT, Xiong Y, Zhong H, Andrzejczyk NE, Zheng C, Schlenk D (2020). Transcriptomic responses of bisphenol S predict involvement of immune function in the cardiotoxicity of early life-stage zebrafish (Danio rerio). Environ Sci Technol.

[25] Kivivirta K, Herbert D, Lange M, Beuerlein K, Altmuller J, Becker A (2019). A protocol for laser microdissection (LMD) followed by transcriptome analysis of plant reproductive tissue in phylogenetically distant angiosperms. Plant Methods.

[26] Marco-Puche G, Lois S, Benitez J, Trivino JC (2019). RNA-Seq perspectives to improve clinical diagnosis. Front Genet.

[27] Thorpe LM, Yuzugullu H, Zhao JJ (2015). PI3K in cancer: divergent roles of isoforms, modes of activation and therapeutic targeting. Nat Rev Cancer.

[28] Kato S, Iida S, Higuchi T, Ishikawa T, Takagi Y, Yasuno M, Enomoto M, Uetake H, Sugihara K (2007). PIK3CA mutation is predictive of poor survival in patients with colorectal cancer. Int J Cancer.

[29] Sartore-Bianchi A, Martini M, Molinari F, Veronese S, Nichelatti M, Artale S, Di Nicolantonio F, Saletti P, De Dosso S, Mazzucchelli L (2009). PIK3CA mutations in colorectal cancer are associated with clinical resistance to EGFR-targeted monoclonal antibodies. Cancer Res.

[30] Zhu YF, Yu BH, Li DL, Ke HL, Guo XZ, Xiao XY (2012). PI3K expression and PIK3CA mutations are related to colorectal cancer metastases. World J Gastroenterol.

[31] Hong DS, Bowles DW, Falchook GS, Messersmith WA, George GC, O'Bryant CL, Vo AC, Klucher K, Herbst RS, Eckhardt SG (2012). A multicenter phase I trial of PX-866, an oral irreversible phosphatidylinositol 3-kinase inhibitor, in patients with advanced solid tumors. Clin Cancer Res.

