# Supplementary material for: Transcriptome profiling and co-expression network analysis of lncRNAs and mRNAs in colorectal cancer by RNA sequencing
Source: BMC Cancer. 2022 Jul 16;22:780. doi: 10.1186/s12885-022-09878-6 (PMC9288709; doi:10.1186/s12885-022-09878-6)
Supplement: Supplementary file 2 — Additional file 2: Table S1. (DOCX 13 kb) [file 12885_2022_9878_MOESM2_ESM.docx]

**Table S1** The information for 6 paired of CRC patients

| Grouping | Stage | Sample id | Sample name | Sample type |
| --- | --- | --- | --- | --- |
| Group All |  |  |  |  |
| Group 1 |  |  |  |  |
| CRC-07 | IIb | s01 | N1 | Normal |
|  |  | s02 | T1 | Tumor |
| CRC-09 | IIb | s03 | N2 | Normal |
|  |  | s04 | T2 | Tumor |
| CRC-11 | IIb | s05 | N3 | Normal |
|  |  | s06 | T3 | Tumor |
| Group 2 |  |  |  |  |
| CRC-08 | IIIb | s07 | N4 | Normal |
|  |  | s08 | T4 | Tumor |
| CRC-30 | IIIb | s09 | N5 | Normal |
|  |  | s10 | T5 | Tumor |
| CRC-35 | IIIb | s11 | N6 | Normal |
|  |  | s12 | T6 | Tumor |
