# Supplementary material for: Transcriptome profiling and co-expression network analysis of lncRNAs and mRNAs in colorectal cancer by RNA sequencing
Source: BMC Cancer. 2022 Jul 16;22:780. doi: 10.1186/s12885-022-09878-6 (PMC9288709; doi:10.1186/s12885-022-09878-6)
Supplement: Supplementary file 3 — Additional file 3: Table S2. (DOCX 13 kb) [file 12885_2022_9878_MOESM3_ESM.docx]

**Table S2** The primer sequences

| Gene Symbols | Location | Primer Sequences |
| --- | --- | --- |
| BBOX1-AS1 | F | 5’-GATGGGCACATTTGGAAGT-3’ |
|  | R | 5’-GGTCAGGGTAACCGTAGCA-3’ |
| MIR503H | F | 5’-CTCCCACCATTTCTTTCC-3’ |
|  | R | 5’-GACCCTGGTTATTGACATCTA-3’ |
| SLCO4A1-AS1 | F | 5’-CACCTAGAGCCTGGTTCCG-3’ |
|  | R | 5’-GCACCGTCTGTTCCTGATT-3’ |
| MAFG-AS1 | F | 5’-GTTCCGTGGTCAGTGAGGGC-3’ |
|  | R | 5’-CCGGTCGTGGAGATGAGTGT-3’ |
| MIR4435-1HG | F | 5’-TGAGAATGAAGGCTGAGGTG-3’ |
|  | R | 5’-TCCCAGGAACTGTGCTGTGAA-3’ |
| DPP10-AS | F | 5’-CAGCCCAGATTCTCCTACC-3’ |
|  | R | 5’-GCTCCCTTCTTCCAAGTGTT-3’ |
| SATB2-AS1 | F | 5’-ATGTGTGCAACACCTTCCAGA-3’ |
|  | R | 5’-GGTGACCAGCTTGGTCTAGG-3’ |
| SEMA6A-AS1 | F | 5’-AGGGTCCTCCTCCCTTCAA-3’ |
|  | R | 5’-GGTGAATTGCCTTGACTGCC-3’ |
| LINC01133 | F | 5’-GGTAGACATCAGTGGTGGTAA-3’ |
|  | R | 5’-AACAAGGACTTTCTGGTGGG-3’ |
| LINC00261 | F | 5’-CTTTGACCCTCCCAAGCATT-3’ |
|  | R | 5’-GTTCACAGACCTCCTTCCCT-3’ |
| GAPDH | F | 5’-CATGTTCGTCATGGGTGTGAACCA-3’ |
|  | R | 5’-AGTGATGGCATGGACTGTGGTCAT-3’ |

**Notes:** The RT-qPCR thermal procedure was as following: 95℃ for 30sec, 40 cycles of 95℃ for 5 sec, 60℃ for 32 sec.
