# Supplementary material for: Transcriptome profiling and co-expression network analysis of lncRNAs and mRNAs in colorectal cancer by RNA sequencing
Source: BMC Cancer. 2022 Jul 16;22:780. doi: 10.1186/s12885-022-09878-6 (PMC9288709; doi:10.1186/s12885-022-09878-6)
Supplement: Supplementary file 4 — Additional file 4: Table S3. (DOCX 15 kb) [file 12885_2022_9878_MOESM4_ESM.docx]

**Table S3** Gene ontology analysis (GO) for lncRNAs-related mRNAs

| GO Term | List hits | List size | Pop hits | Pop size | Enrichment  Fold | FDR |
| --- | --- | --- | --- | --- | --- | --- |
| GO:0006635~fatty acid beta-oxidation | 11 | 628 | 39 | 16412 | 7.37 | 2.56E-05 |
| GO:0044281~small molecule metabolic process | 104 | 628 | 1706 | 16412 | 1.59 | 2.73E-05 |
| GO:0001558~regulation of cell growth | 12 | 628 | 74 | 16412 | 4.24 | 1.94E-03 |
| GO:0007165~signal transduction | 63 | 628 | 1036 | 16412 | 1.59 | 5.11E-03 |
| GO:0050871~positive regulation of B cell activation | 7 | 628 | 25 | 16412 | 7.32 | 5.14E-03 |
| GO:0015701~bicarbonate transport | 8 | 628 | 40 | 16412 | 5.23 | 1.27E-02 |
| GO:0030198~extracellular matrix organization | 25 | 628 | 310 | 16412 | 2.11 | 1.53E-02 |
| GO:0010628~positive regulation of gene expression | 20 | 628 | 232 | 16412 | 2.25 | 2.66E-02 |
| GO:0071456~cellular response to hypoxia | 13 | 628 | 119 | 16412 | 2.85 | 3.49E-02 |
| GO:0007507~heart development | 17 | 628 | 185 | 16412 | 2.40 | 3.50E-02 |
| GO:0090280~positive regulation of calcium ion import | 5 | 628 | 15 | 16412 | 8.71 | 3.61E-02 |
| GO:1902043~positive regulation of extrinsic apoptotic signaling pathway via death domain receptors | 5 | 628 | 15 | 16412 | 8.71 | 3.61E-02 |
| GO:0006898~receptor-mediated endocytosis | 15 | 628 | 152 | 16412 | 2.58 | 3.65E-02 |
| GO:0001501~skeletal system development | 14 | 628 | 136 | 16412 | 2.69 | 3.74E-02 |
| GO:0030574~collagen catabolic process | 10 | 628 | 75 | 16412 | 3.48 | 3.80E-02 |
| GO:0006911~phagocytosis, engulfment | 7 | 628 | 36 | 16412 | 5.08 | 3.89E-02 |

**Notes:** List hits: numbers of differentially expressed genes in the pathway. List size: total numbers of differentially expressed genes. Pop hits: numbers of background genes in the pathway. Pop size: total numbers of background genes. Enrichment Fold: Multiple of enrichment, (List hits/ List size) / (Pop hits/ Pop size). FDR: false discovery rate.
