# Supplementary material for: Transcriptome profiling and co-expression network analysis of lncRNAs and mRNAs in colorectal cancer by RNA sequencing
Source: BMC Cancer. 2022 Jul 16;22:780. doi: 10.1186/s12885-022-09878-6 (PMC9288709; doi:10.1186/s12885-022-09878-6)
Supplement: Supplementary file 5 — Additional file 5: Table S4. (DOCX 14 kb) [file 12885_2022_9878_MOESM5_ESM.docx]

**Table S4** Kyoto Encyclopedia of Genes and Genomes pathway (KEGG) analysis for lncRNAs-related mRNAs

| KEGG PATHWAY | List  hits | List  size | Pop  hits | Pop  size | Enrichment  Fold | FDR |
| --- | --- | --- | --- | --- | --- | --- |
| hsa00071:Fatty acid degradation | 10 | 287 | 44 | 6891 | 5.46 | 8.30E-04 |
| hsa00280:Valine, leucine and isoleucine degradation | 10 | 287 | 47 | 6891 | 5.11 | 1.43E-03 |
| hsa00650:Butanoate metabolism | 7 | 287 | 27 | 6891 | 6.22 | 8.95E-03 |
| hsa01100:Metabolic pathways | 71 | 287 | 1217 | 6891 | 1.40 | 2.41E-02 |
| hsa04512:ECM-receptor interaction | 11 | 287 | 87 | 6891 | 3.04 | 3.93E-02 |
| hsa01212:Fatty acid metabolism | 8 | 287 | 48 | 6891 | 4.00 | 4.31E-02 |
| hsa04151:PI3K-Akt signaling pathway | 26 | 287 | 345 | 6891 | 1.81 | 5.59E-02 |
| hsa00910:Nitrogen metabolism | 5 | 287 | 17 | 6891 | 7.06 | 5.74E-02 |
| hsa00640:Propanoate metabolism | 6 | 287 | 27 | 6891 | 5.34 | 5.76E-02 |

**Notes:** List hits: numbers of differentially expressed genes in the pathway. List size: total numbers of differentially expressed genes. Pop hits: numbers of background genes in the pathway. Pop size: total numbers of background genes. Enrichment Fold: Multiple of enrichment, (List hits/ List size) / (Pop hits/ Pop size). FDR: false discovery rate.
